# Beyond Fitzpatrick: automated artificial intelligence-based skin tone analysis in dermatological patients

**DOI:** 10.1038/s41746-025-01770-4

**Published:** 2025-06-20

**Authors:** Paul Ulrich, Alexander Zink, Tilo Biedermann, Sebastian Sitaru

**Affiliations:** 1 Technical University of Munich, TUM School of Medicine and Health, Department of Dermatology and Allergy, Munich, Germany; 2 Department of Dermatology and Venereology, University Hospital Regensburg, Regensburg, Germany

**Keywords:** Medical research, Computer science, Health care, Skin manifestations

## Abstract

Human skin tone is influenced by genetic, environmental, and cultural factors and plays a key role in dermatology due to variation in disease presentation across skin tones. The widely used Fitzpatrick scale, based on UV response, classifies only a small number of skin types, limiting its ability to capture the full diversity of skin tones. This study introduces an algorithm for automated skin tone assessment by calculating the Individual Typology Angle (ITA) from CIELAB color values using DensePose and OpenFace. ITA values are mapped to both Fitzpatrick and Monk skin tone scales. Validation on 3D body scans and AI-generated images showed high agreement with Monk classifications but less consistent alignment with Fitzpatrick types. Despite class imbalance, the algorithm reliably classifies skin tone to the Monk scale and holds potential for applications in teledermatology, clinical research, and personalized medicine. Further research is warranted to externally validate our algorithm.

## Introduction

Human skin tone is a complex trait influenced by genetic, environmental, and cultural factors. Depending on the density and distribution of melanocytes in the basal epidermis, it manifests along a continuum which presents challenges for standardized assessment in dermatology^1^.

While recognizing this continuum, classification systems are indispensable for clinical practice as an important tool in diagnosis, treatment planning and evaluation, as well as for epidemiological studies^2^.

The Fitzpatrick scale, currently the gold standard for skin tone classification in the medical community^3^, was initially devised to categorize patients´ response to UV radiation^4^. Originating with four types tailored for fairer skin, it later expanded to include six types, with two darker skin types. Later, photospectrometry and colorimetry were introduced into research and to a lesser extend the clinical practice^5^. Researchers found a way to determine the Individual Typology Angle (ITA), which serves as a pivotal metric in objective skin tone assessment, offering a quantitative measure of an individual’s skin color^6^. The ITA is calculated based on the mean CIELAB-color from the examined area. The CIELAB color space, also known as Lab color space, is a three-dimensional color model designed to approximate human perception of color. Developed by the International Commission on Illumination (CIE), it consists of three coordinates: L* for lightness, a* for the green-red axis, and b* for the blue-yellow axis. In this color space, L* represents the lightness of a color, ranging from 0 for black to 100 for white. The a* and b* coordinates represent the chromaticity of the color, with positive values indicating the presence of red or yellow hues and negative values indicating green or blue hues^7^.

The ITA can be calculated from these values by using the following Eq. (1)^6^:

1 $$\mathrm{ITA}=[\arctan (({{\rm{L}}}^{* }-50)/{{\rm{b}}}^{* })]\,{\rm{x}}\,180/\mathrm{pi}$$

The ITA serves as a valuable complement to classification systems, offering a continuous measure that acknowledges the nuanced nature of skin color as it includes many variations in skin pigmentation, providing insights into the degree of melanin distribution and overall skin tone. By quantifying subtle changes in pigmentation, the ITA facilitates more precise and comprehensive skin tone assessment, enhancing diagnostic accuracy and treatment planning in dermatological practice. Additionally, its objective nature makes it particularly well-suited for integration into machine learning algorithms, enabling automated and standardized skin tone evaluation in diverse clinical settings.

For clinical classification, the continuous ITA value is often divided into 6 classes, thus similar to the Fitzpatrick skin types, but not actually interchangeable^8,9^.

Recent work has highlighted the shortcomings of these existing systems in both inclusivity and accuracy, suggesting that traditional metrics, such as the Fitzpatrick scale, are not only limited in their scientific grounding but also susceptible to inter-rater variability and racial bias, especially in automated image processing contexts^10^. In response to this, the Monk Skin Tone Scale has been developed as a 10-tone, open-source scale aimed at improving inclusivity in skin tone classification across technological and clinical domains^11^. Unlike previous scales such as the Fitzpatrick phototype, which was originally designed to predict UV sensitivity and has shown bias toward lighter skin types, the Monk scale explicitly aims to represent the global spectrum of skin tones with greater equity and usability.

This novel scale describes 10 skin tone types, striking a balance between inclusivity and differentiation while still ensuring reproducibility and comprehensibility, making it suitable for diverse real-world applications such as dermatology, computer vision, and fairness auditing in AI models. By providing a more reliable and representative framework, it could partly overcome the limitations of existing classification systems and lead to better representation across diverse populations^12^.

Skin tone assessment is an important part of dermatological evaluations as dermatoses show diverse prevalence across different skin tone and can look drastically differently in skin of color than in lighter skin tones^13^. Having an objective tool to quantify skin tone and changes of skin tone on images while also being translatable into classes easily comprehensible for human examiners could prove as a very valuable asset, particularly in the burgeoning field of telemedicine and for monitoring disease progression during follow-up examinations^14^. Furthermore, the Monk Skin Tone Scale presents new possible paths for research, e.g. for investigations into the prevalence of dermatological and other conditions across different skin tone groups using large scale datasets.

In this context, the development of a digital algorithm capable of objectively assessing skin tone, as showcased in this study, holds promise, especially when paired with a classification system that focuses on skin tone instead of how an individual´s skin reacts to UV exposure. To our knowledge, there is currently no totally automatic application for this use case. Similar approaches like the 2023 published Skin Analyzer^15^ depend on users manually selecting the skin area in question, therefore limiting the scalability. Our fully automatic approach could streamline skin tone assessment by making them faster and more objective, especially when handling large datasets, as manual annotation is very time consuming, and contribute to advancing our understanding of skin-related conditions in diverse populations. Additionally, it opens ways for exploring associations between skin tone and disease prevalence in large datasets, potentially shedding light on disparities in healthcare outcomes.

## Results

**Fig. 1:**
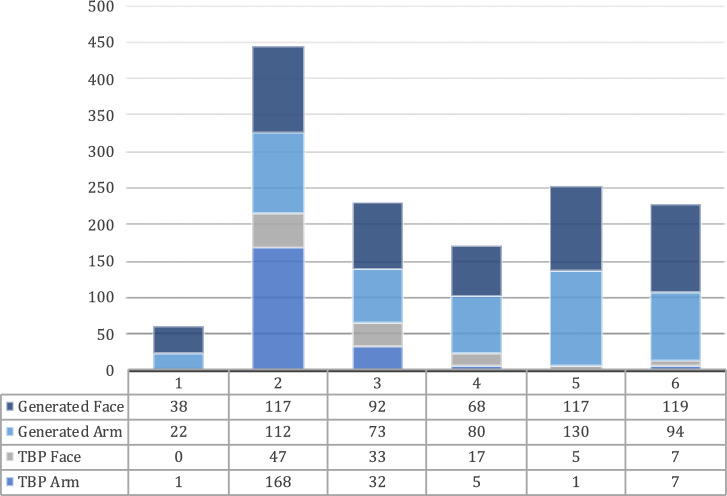
Image count by Fitzpatrick skin type. The figure shows the image count per Fitzpatrick Skin Type, categorized by clinical vs. AI-generated origin and body region assessed.

**Fig. 2:**
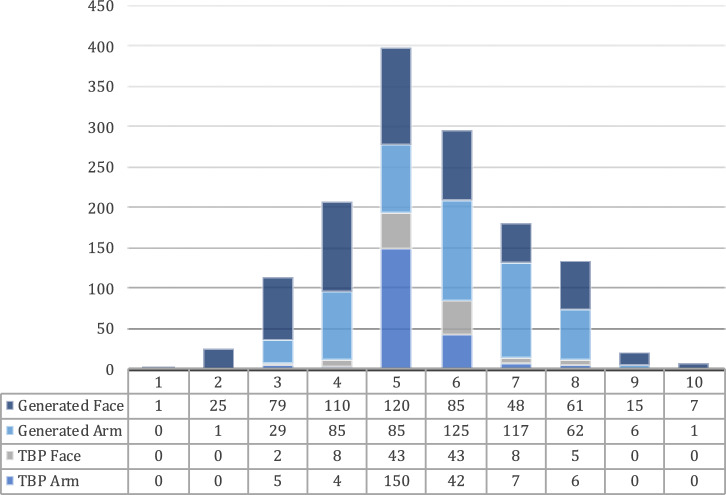
Image count by Monk skin tone. The figure shows the image count per Monk Skin Tone, categorized by clinical vs. AI-generated origin and body region assessed.

From 258 3D TBP scans screened, 214 scans yielding 214 images of the whole body, and 109 images of the face, of 214 unique patients, were considered for the further study. The reasons for images to be not considered included dermatoses with erythema, or tattoos at the relevant body sites. In addition, 551 unique AI-generated images depicting the whole body from the front including face and arms were considered. Here, *n* = 50 images could not be used for pixel detection on the forearm because of clothing or body posture. The distribution of images in each category by Fitzpatrick and Monk skin tone are shown in Figs. 1 and 2, respectively.

### Algorithm accuracy for skin tone classification

The algorithm classified clinical images to the Monk Skin Tone Scale with 89-92% accuracy and AI-generated images with 84-89% accuracy. For the classification to the Fitzpatrick Scale, accuracies of 20% for clinical images showing the face and 0.5% for clinical images showing the arms, were achieved. In AI-generated images, the model reached an accuracy of 10% for the face and 67% for the arm when working with the Fitzpatrick Scale (Table 1).

Regarding the Monk Scale, the algorithm achieves a higher accuracy and sufficient accuracy for real images on both face and arm compared to its performance for generated images, where nonetheless also high levels of accuracy and sufficient accuracy could be achieved. Balanced accuracy remained consistently high across both body regions in clinical images, reaching between 68% for the face and 66% for the arm. Balanced accuracy for generated images achieved similar numbers, ranging from 58% for the face to 75% for the arm (Table 1).

Working with the Fitzpatrick Scale, there has been a lower accuracy for real images compared to generated images, especially for arm regions. The Fitzpatrick Scale also showed lower and more variable balanced accuracy values, with 44% for clinical facial images and 17% for clinical images of the arm. Working with generated images, the balanced accuracies also deviated further, as the algorithm reaches up to 65% when classifying the arm, while only 19% working with the face (Table 1).

When analyzing the per-class accuracies for both Fitzpatrick and Monk scale, it becomes evident that the algorithm accuracy varies widely across the classes (Fitzpatrick: 12.39% - 85.25%; Monk: 0 – 99.66%; supplementary tables 2 and 3).

**Table 1:** Accuracy

Region	Scale	Accuracy	Sufficient Accuracy	Balanced Accuracy
Clinical Images				
Face	Monk	88.99%	98.17%	67.59%
Arm	Monk	91.59%	97.66%	65.67%
Face	Fitzpatrick	20.18%	42.20%	44.26%
Arm	Fitzpatrick	0.47%	6.07%	16.67%
AI-generated Images				
Face	Monk	84.21%	98.55%	57.87%
Arm	Monk	89.04%	98.63%	74.88%
Face	Fitzpatrick	10.34%	58.26%	19.38%
Arm	Fitzpatrick	66.73%	91.59%	65.25%

Table 1 compares accuracy, sufficient accuracy, and balanced accuracy of skin tone classification by region and scale for both clinical and AI-generated images.

### Influence of image type and body site on accuracy

To examine the differences in accuracy between the four groups stemming from the combinations of test site and image type (arm, face, generated arm and generated face) we performed Mann-Whitney U Tests.

Regarding the accuracy for clinical images by body site, the results of the Mann-Whitney U Tests show that the differences are not significant for the Monk Scale (*p* = 0.448) while being highly significant for the Fitzpatrick Scale (*p* < 0.001).

For generated images, the results show that the differences in accuracy by body site are significant for the Monk Scale (*p* = 0.021) and highly significant for the Fitzpatrick Scale (*p* < 0.001).

Concerning the differences in accuracy by lighter and darker skin tones, the Mann-Whitney U Tests show, that the differences are not significant for the Monk Scale (*p* = 0.710) while being highly significant for the Fitzpatrick Scale (*p* < 0.001).

## Discussion

In our study, we created an algorithm for the objective and fully automatic classification of dermatological images to skin tones. While for the Fitzpatrick Scale, very low accuracies of 0–20% were observed, the algorithm performed well when using the Monk Scale as a comparator (89–92% accuracy). Furthermore, the algorithm performed slightly better on AI-generated (10%–89% accuracy) than real-life clinical images (0%-92% accuracy). The highest accuracies were observed for skin tone evaluation of the arm in AI-generated images (67–89%) and differences in accuracy were statistically significant for most subgroup analyses of image type and body site, indicating that these parameters have influence on how well the algorithm performs. Importantly, accuracies for the Monk Scale were also stable across lighter (Monk 1-5) and darker (Monk 6-10) Skin Tones while the accuracies across the corresponding Fitzpatrick skin types (1–3 and 4–6) showed statistically significant differences. Similarly, balanced accuracies were overall higher and more stable for the Monk scale (58%–75%) compared to the Fitzpatrick scale (17%–65%), indicating more consistent classification performance across skin tone categories when using the Monk framework instead of Fitzpatrick Skin Type. One possible explanation for the gap between accuracy and balanced accuracy could be the class imbalance of the dataset, leading to a wide range of accuracies in the individual classes.

AI-generated images consistently outperformed clinical images in terms of accuracy. This might be due to the fact that generated images are standardized in lighting, pose, and quality, whereas clinical images often exhibit variability in conditions such as lighting, posture, and skin appearance. Even the 3D TBP images we used, where every patient is photographed in a standard pose, show slight differences in these aspects and therefore are more challenging for our model to classify. A notable discrepancy was observed between face and arm classification accuracy when comparing the Monk and Fitzpatrick scales. While the Monk scale achieved relatively high and consistent accuracy for both regions, the Fitzpatrick scale showed a much larger drop in accuracy for facial images. This difference may be attributed to multiple factors: First, the Fitzpatrick classification incorporates originally additional features such as eye color, hair color, and tanning behavior, which cannot be assessed from ITA values alone, thereby limiting the scale’s applicability in ITA-based assessments. Second, facial regions often exhibit greater variability in lighting, skin texture, and presence of makeup or facial hair, all of which may interfere with accurate ITA-based classification. In contrast, the forearm typically offers a more uniform and unobstructed skin surface for color sampling, leading to better performance, particularly for structured algorithms like the one used for our model.

Interestingly, the Monk Scale maintained consistent accuracy across lighter and darker skin tones, unlike the Fitzpatrick Scale, which showed better performance for darker tones. This suggests that the Monk Scale’s design is in fact better suited for inclusive and detailed classification.

Several additional factors may have influenced the observed performance differences of the algorithm. The algorithm’s reliance on ITA values may still oversimplify the complexity of real-world skin tone variations, as it does not account for texture or pigmentation irregularities that could introduce noise into its predictions. On the other hand, synthetic images may lack these natural imperfections or variations present in real skin, as well as influences stemming from underlying skin conditions of the patients, making them easier for the algorithm to classify. Finally, the segmentation accuracy of DensePose and OpenFace could vary between clinical and synthetic images. Any mislabeling of regions or inclusion of non-skin areas during segmentation could distort the ITA values and lead to errors in classification. However, both algorithms were developed using real life images, which mitigates this limitation to a degree^16,17^.

It is important to acknowledge that skin tone exists along a continuum, rather than as discrete categories, and skin tone description via the Individual Typology Angle (ITA) instead of continuously using a classification system could be a possible way to translate this fact into dermatological practice. It also considers how future treatments and diagnostics trend towards more personalized medicine^18^. Instead of falling under one of six categories, patients could receive their individual ITA based off a standardized imaging process. An approach as such could provide a nuanced perspective on skin tone assessment, particularly in clinical contexts, where a deeper understanding of skin pigmentation and changes of pigmentation are important factors for accurate diagnosis, treatment planning and control^19,20^. However, there will still be applications where classifications are needed. In these cases, the classification system should both be as reliable and inclusive as possible. The Fitzpatrick Skin Type classification was originally designed for evaluating photosensitivity and not necessarily skin tone per se, and includes hair color, eye color, and presence of freckles, amongst other factors^4^. Despite this fact, in our experience it is often used clinically as a sole descriptor of the skin tone. Our study reinforces the “intended” use of the Monk Scale, rather than Fitzpatrick Scale, for the classification of skin tones, as the Monk Scale has outperformed the Fitzpatrick Scale in terms of mapping ITA values to a skin tone class.

Our algorithm opens up new possibilities for clinical and research applications. By providing objective and standardized skin tone assessments, it can support more accurate diagnoses and treatment recommendations, especially in remote settings where access to dermatological expertise may be limited. Therefore, one potential use case lies within the field of dermatology, as remote consultations and hybrid care approaches turn increasingly more popular and common^21^.

Additionally, the algorithm’s ability to assess skin tone in real-life clinical images holds promise for large-scale epidemiological studies, enabling researchers to investigate links between skin tone and various dermatological conditions or disease outcomes, even when handling large amounts of data^22^. Researchers might also use the algorithm to analyze large-scale datasets of clinical images to identify patterns and trends in skin tone variations across different demographics, geographic regions, and ethnicities. This could lead to insights into the prevalence and distribution of dermatological conditions, and potentially even help bridge existing health gaps regarding diagnosis and treatment between patients with fairer and darker skin tones^23–26^.

Furthermore, the incorporation of machine learning algorithms in dermatological practice has the potential to improve patient care and outcomes^27,28^. With ongoing advancements in technology and algorithm refinement, future iterations of our algorithm could incorporate additional parameters and features, such as texture analysis or lesion assessment, especially when paired with the ability to segment lesions automatically.

Our system has shown to work well when correlated with the Monk Skin Tone Scale. However, when working with the Fitzpatrick Scale, the accuracy is not good enough for it to be considered a tool to automate classification of images to a Fitzpatrick Skin Type. As previously described, this might be due to the automatic inclusion of other factors regarding Fitzpatrick Skin Type when dermatologically trained researchers diagnose it. Possibly, an end-to-end classification model e.g., using artificial neural networks, might perform better for this task. As already mentioned, other studies have also shown that previously stated thresholds to convert ITA values to Fitzpatrick phototypes do not correlate well enough with real life Fitzpatrick phototypes.

Regarding arm segmentation, ITA values were extracted from DensePose-generated masks that label the left and right lower arm regions. While this approach ensures a standardized anatomical reference, it limits the area used for color extraction to a relatively small fraction of the forearm. This restricted sampling area may introduce variability in cases with non-standard body postures or partial occlusion. In future iterations, more robust alternatives such as computing a bounding rectangle around the detected forearm region or expanding the segmentation mask to include a larger area could be conceived, thereby increasing the number of pixels used for ITA calculation and improving classification stability.

Furthermore, while images portraying all 10 Monk Skin Tones and all 6 Fitzpatrick Skin Types have been part of this study, there was a class imbalance towards more intermediate skin tones. Further research is warranted to externally validate the model and to better validate it for skin tones with very low or very high ITA values.

In summary, our AI-based, fully automatic algorithm can classify skin tones in clinical and synthetically generated skin images, with different performance between the image types and Fitzpatrick and Monk Skin Tone Scales. While for classifying to the Monk Skin Tone Scale, promising accuracies of 89-92% were observed, for the Fitzpatrick Scale no clinically useful classification was possible. Therefore, our tool could provide the basis for one-click, automated evaluation of skin tones with future potential especially in teledermatology, personalized medicine and clinical studies. On the other hand, our results also show that the Monk Skin Tone Scale shows promise as a more inclusive and reliable classification system for skin tone assessment, warranting further investigation and adoption in clinical practice and research.

## Methods

### Data sources

Two data sources were used: first, routine three-dimensional total body photography scans (3D TBP) of patients at the Department of Dermatology and Allergy, TUM University Hospital rechts der Isar, Munich, and second, generated images of persons using the artificial intelligence-based tool Generated Photos and their pre-generated academic dataset^29^. These images were used to extend the rather small clinical dataset, especially for darker skin types, which are not prevalent at our department. From the 3D TBP scans, images are routinely rendered for 2D compatibility purposes, showing the front, back and face of the patient. The rendered images of the face, as well as the back of the patient, were used for this study. Images showing (sub-)erythroderma, were excluded, since the algorithm relies on ITA values calculated from CIELAB color space and the presence of erythema could lead to inaccurate skin tone measurements. While clinicians can accurately assess the underlying skin type of erythematous skin despite such changes - based on context, medical history, or unaffected areas - our fully automated approach cannot currently make such adjustments.

### Artificial intelligence-based analysis of arm and face color

**Fig. 3:**
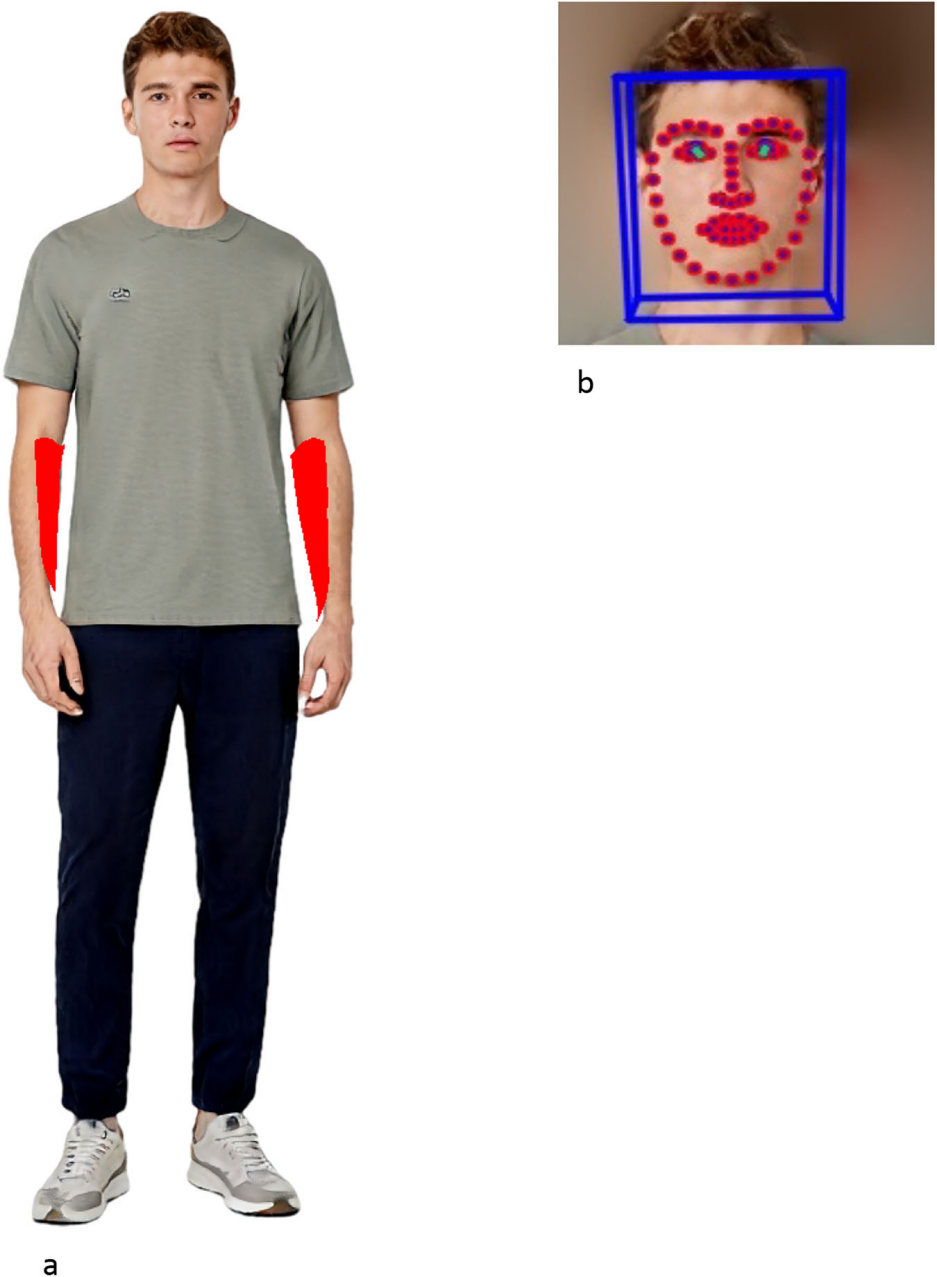
Pixel detection. **a** Pixel detection using the Detectron framework on the forearm. Red – segmentation mask for the forearm, whose pixels were used for skin type evaluation of the arm. AI-generated image used with permission from Generated Photos^29^. **b** Pixel detection using the OpenFace framework. Blue – bounding box of the recognized face. Red – landmark pixels. The pixels on the nose were used for skin type evaluation of the face. AI-generated image used with permission from Generated Photos^29^.

To obtain the pixels making up the lower arm, the DensePose algorithm with the configuration densepose_rcnn_R_101_FPN_s1x and model ID c6ab63 was used^16^. The RGB values of pixels of segmentation labels 19 and 20, corresponding to the left and right lower arm, were obtained (Fig. 3a). The RGB values were then converted into CIELAB values. From the CIELAB values, each pixel’s individual topology angle (ITA) as a measure of skin type was calculated as described before^7^. The mean of these values for one image (patient) was defined as the ITA value of the arms for that patient.

To obtain the pixels on the face, the OpenFace framework (v. 2.2.0) was used^17^. The RGB pixel values of landmarks 27, 28, 29, 30, corresponding to the pixels on the back of the nose, were converted to ITA values as described above (Fig. 3b). The nasal bridge was chosen as the region for facial ITA calculation due to being centrally located, generally free from facial hair, and less prone to occlusion or cosmetic alteration compared to other facial areas. Additionally, the nasal region is typically well-exposed and uniformly illuminated in both clinical and synthetic images. The mean of these ITA pixel values for one image (patient) was defined as the ITA value of the face for that patient.

ITA values were then mapped to Fitzpatrick skin types and Monk scale skin types for the arm and the face using Tables 1 and 2. For Fitzpatrick phototypes, such a table has already been published and used and we opted to use the same values^7,15^.

**Table 2:** Correlation Table Monk

Monk	ITA
1	(100.0, 81.62)
2	(81.62, 75.99)
3	(75.99, 68.24)
4	(68.24, 57.53)
5	(57.53, 30.61)
6	(30.61, −4.63)
7	(−4.63, −37.77)
8	(−37.77, −66.87)
9	(−66.87, −81.33)
10	(−81.33, −100.00)

Table 2 shows the newly developed correlation table for the Monk Skin Tone Scale. Each Monk tone (1–10) corresponds to a defined ITA range, enabling automated skin tone categorization from CIELAB measurements.

**Fig. 4:**
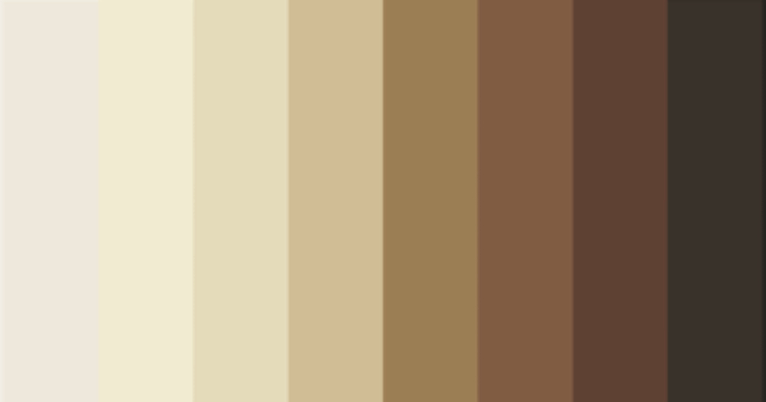
Visualization of the Monk skin tone scale. The ten skin tone categories defined by the Monk Scale are presented from lightest (1) to darkest (10). Image source: https://skintone.google^30^.

For the newly developed Monk Scale, to our knowledge, a correlation table has not been published yet. To establish a mapping between ITA values and the Monk Skin Tone Scale, we calculated the ITA for each of the ten reference skin tones defined in the Monk scale (Fig. 4). Thresholds between tones were defined as the mean ITA values between adjacent categories, resulting in ten ITA intervals corresponding to Monk tones 1 through 10. This correlation table enables the algorithm to assign ITA-derived values directly to Monk skin tone categories in a transparent and reproducible manner (Table 2).

To compare both skin tone scales, the results were then matched to the respective skin types annotated by 3 dermatologically trained researchers and the differences in agreement for both types of scales were examined. We used overall accuracy as the primary metric to evaluate the performance of our algorithm. This was based on the practical relevance of accuracy in clinical applications, where each individual case (i.e., each image) carries equal importance, regardless of its underlying class. To ensure both reliable evaluation and clinical applicability, we define accuracy with a 10% deviation margin. This tolerance accounts for minor uncertainties arising from external factors such as lighting conditions or labeling variability, which are common in real-world settings. At the same time, it reflects the structural properties of the Monk scale, whose ten classes each span a relatively narrow ITA range, making small deviations more impactful. In contrast, the Fitzpatrick scale consists of fewer, broader categories, where a single class covers a wider ITA interval. Accuracy was then calculated by the following Eq. (2):

2 $${\rm{Accuracy}}=\frac{{\bf{correct\; classifications}}}{{\bf{total\; number\; of\; images}}}$$

To complement this analysis, we additionally report sufficient accuracy, which allows a 20% margin of error, approximately equivalent to a one-class shift on the Fitzpatrick scale. We also report the balanced accuracy, which is computed as the average of the recall of each skin tone class, using the same 10% deviation threshold as for accuracy. These definitions ensure a realistic, robust, and practical assessment of the algorithm’s skin tone classification and allow for better comparability with dermatological assessments, as especially for the Monk Scale, deviations by one step are well within the range of outcomes in clinical practice.

### Validation of the algorithm performance

Two digital dermatology researchers trained in skin type analysis each annotated the validation dataset consisting of 214 full body images from 3D TBP, 109 face images from 3D TBP scans, 511 AI-generated full body images regarding the arm and 551 AI-generated images regarding the face, recording the Fitzpatrick and Monk scale (defined as the gold standard). Annotation differences were resolved by discussion. The output of the fully automatic algorithm described before was compared to this annotation yielding % accuracy values. Balanced accuracies were calculated using R v.4.4.1.

### Data access, cleaning and linkage

The investigators had full access to the database population used to create the study population. No data cleaning and/or linkage to other data sources was performed. The generated images are part of the Academic Dataset by Generated Photos and are free for non-commercial use and publication.

### Statistical analysis

Descriptive statistics were generated for all categories. Interrater reliability regarding the dermatologists was tested for both the Monk and the Fitzpatrick Scale using Fleiss´ Kappa. Differences between anatomical locations and clinical and generated images were tested with a Mann-Whitney U Test.

IBM SPSS Statistics for Windows, Version 29.0 (IBM Corp, Armonk, NY) was used for statistical analysis.

### Data protection

Local legislation (Bavarian hospital law/BayKrG, art. 4, par. 27) permits the use of routine patient data for research purposes, therefore no informed consent and/or IRB approval was needed. Patient data was processed locally and not transmitted to any third-party service or machine. All images shown in this publication are AI-generated. No identifiable patient data is shown.

## Supplementary information

Supplementary information

## Data Availability

The original data (patient pictures and generated images) cannot be provided online due to privacy concerns.

## Code availability

The Python scripts used to pre-process the data, train the network, and validate it can be found on GitHub: https://github.com/ssitaru/beyond-fitzpatrick.
