# Supplementary material for: Beyond Fitzpatrick: automated artificial intelligence-based skin tone analysis in dermatological patients
Source: NPJ Digit Med. 2025 Jun 20;8:378. doi: 10.1038/s41746-025-01770-4 (PMC12179258; doi:10.1038/s41746-025-01770-4)
Supplement: Supplementary file 1 — Supplementary information [file 41746_2025_1770_MOESM1_ESM.pdf]

Supplementary Information

Supplementary Tables

Supplementary Table 1: Correlation Table Fitzpatrick (Chardon et al)

Fitzpatrick	ITA
1	(100.00, 55.00)
2	(55.00, 41.00)
3	(41.00, 28.00)
4	(28.00, 10.00)
5	(10.00, -30.00)
6	(-30.00, -100.00)

Supplementary Table 2: Accuracy per Fitzpatrick Skin Type

Fitzpatrick	Accuracy Arm	Sufficient Accuracy Arm	Accuracy Face	Sufficient Accuracy Face
Clinical Images				
1	0%	0%	-	-
2	0%	0%	2.13%	10.64%
3	0%	0%	3.03%	30.30%
4	0%	100%	64.71%	100.00%
5	100%	100%	80.00%	100.00%
6	0%	100%	71.43%	100.00%
AI-generated Images				
1	63.64%	90.91%	100.00%	100.00%
2	46.43%	74.11%	1.71%	100.00%
3	56.16%	89.04%	1.09%	27.17%
4	58.75%	96.25%	0.00%	14.71%
5	84.62%	99.23%	2.56%	35.90%
6	81.91%	100.00%	10.92%	74.79%

Supplementary Table 3: Accuracy per Monk Skin Tone

Monk	Accuracy Arm	Sufficient Accuracy Arm	Accuracy Face	Sufficient Accuracy Face
Clinical Images				
1	-	-	-	-
2	-	-	-	-
3	0.00%	0.00%	0.00%	0.00%
4	0.00%	100.00%	12.50%	100.00%
5	94.00%	100.00%	93.02%	100.00%
6	100.00%	100.00%	100.00%	100.00%
7	100.00%	100.00%	100.00%	100.00%
8	100.00%	100.00%	100.00%	100.00%
9	-	-	-	-
10	-	-	-	-
AI-generated Images				
1	-	-	0.00%	100.00%
2	0.00%	0.00%	64.00%	100.00%
3	6.90%	82.76%	97.47%	100.00%
4	71.76%	98.82%	100.00%	100.00%
5	97.65%	100.00%	100.00%	100.00%
6	99.20%	100.00%	100.00%	100.00%
7	100.00%	100.00%	95.83%	100.00%
8	98.39%	100.00%	14.75%	100.00%
9	100.00%	100.00%	6.67%	60.00%
10	100.00%	100.00%	0.00%	71.43%

Supplementary Table 4: Accuracy per Monk Skin Tone

Monk	Recall Accuracy	Recall Sufficient Accuracy
1	0.00%	100.00%
2	61.54%	96.15%
3	68.70%	89.57%
4	83.09%	99.52%
5	96.48%	100.00%
6	99.66%	100.00%
7	98.89%	100.00%
8	60.45%	100.00%
9	33.33%	71.43%
10	12.50%	75.00%

Supplementary Table 5: Accuracy per Fitzpatrick Skin Type

Fitzpatrick	Recall Accuracy	Recall Sufficient Accuracy
1	85.25%	95.08%
2	12.39%	46.17%
3	18.70%	44.35%
4	34.12%	64.12%
5	46.64%	69.96%
6	41.85%	86.78%
